# Septal rhinoscleroma

**DOI:** 10.4103/0970-0358.73465

**Published:** 2010

**Authors:** Mohamed A. Shoeib

**Affiliations:** Department of Plastic Surgery, Sohag Faculty of Medicine, Sohag, Egypt - 82542

**Keywords:** Rhinoscleroma, nasal septum, scleroma

## Abstract

Rhinoscleroma is a chronic granulomatous condition of the nose and other structures of the upper respiratory tract. Infection by the bacterium *Klebsiella rhinoscleromatis* is said to be the cause. A female patient aged 45 years, presented with a past history of trauma to the nose and swelling on her nose since last 1 year. There was nasal asymmetry and internal nasal examination showed a septal swelling protruding to the right nasal cavity with hypertrophied nasal mucosa and inferior turbinate. Open tip rhinoplasty approach was used to excise the mass, which examined pathologically revealing a rhinoscleroma, fibrotic infiltrative stage.

## INTRODUCTION

Rhinoscleroma is a chronic granulomatous condition of the nose and other structures of the upper respiratory tract. Rhinoscleroma is a result of infection by the bacterium *Klebsiella rhinoscleromatis*. The Polish surgeon Johann von Mikulich in Wroclaw described its histological features in 1877. and von Frisch identified the organism in 1882. In 1932, Belinov proposed the use of the term scleroma respiratorium because the pathological process in rhinosclerosis may involve not only the upper but also the lower airways. In 1961, Steffen and Smith demonstrated that *K rhinoscleromatis* conformed to Koch’s postulates and that it was an aetiologic factor in the inflammatory changes typical of scleroma. The occurrence of familial disease suggests that genetic control of the host response to *K rhinoscleromatis* may be an important factor in endemic areas.[1,2]

Rhinoscleroma is contracted by means of direct inhalation of droplets or contaminated material. The disease probably begins in areas of epithelial transition such as the vestibule of the nose, the subglottic area of the larynx, or the area between the nasopharynx and oropharynx.[3,4]

## CASE REPORT

A female patient, aged 45 years, presented to the outpatient clinic of Sohag Teaching Hospital with a past history of nasal trauma and swelling in the nose since last one year. The swelling was of not increasing in size and was associated with unilateral nasal obstruction, but no episatxis, nasal dryness, crustation, or other nasal symptoms. No other respiratory symptoms or past history of nasal symptoms were present.

**Figure 1:**
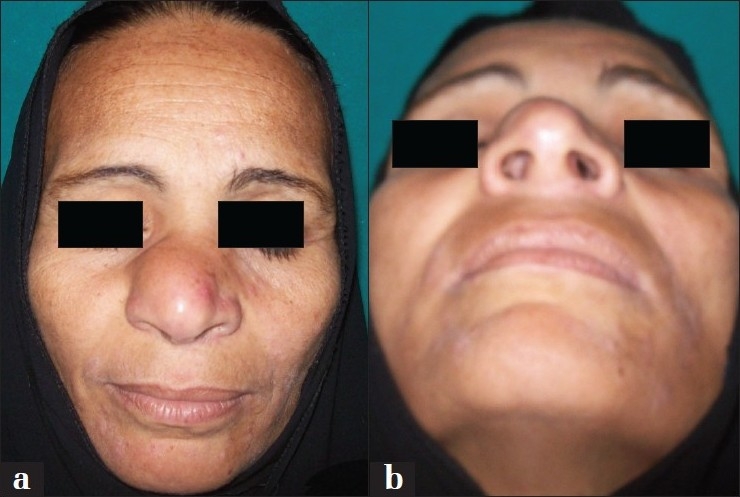
(a and b) Preoperative views of the patient, showing the swelling both internally and externally

General examination showed no fever, no hypertension or diabetes mellitus. On local examination:-, the swelling was in the right side and in the tip of the nose, of 3 × 5 cm in size, firm in consistency, with an ill -defined border and disfiguring the shape of the nose [Figure 1]. Internal nasal examination revealed a septal swelling protruding to the right nasal cavity with hypertrophied nasal mucosa and inferior turbinate. There was no cervical lymph node enlargement.

Because the complaint of the patient was nasal disfigurement, and the nature of the swelling was not known, medical treatment was not considered. The presentation was not conclusive of infectious disease, so the patient was prepared for excisional biopsy.

**Figure 2:**
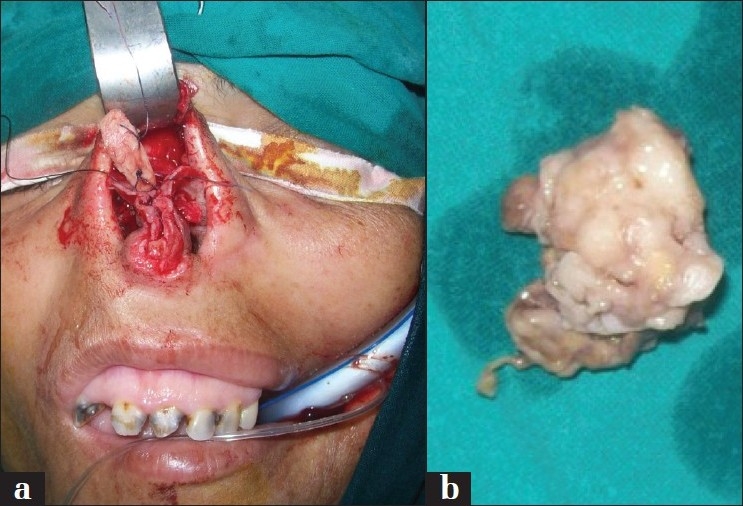
Intraoperative pictures of the swelling arising from the septum (a) and after excision (b)

Through an open rhinoplasty incision a surgical excision was planned. The swelling was originating from the septum and the right upper lateral cartilage with a -well-defined plane of cleavage as shown in Figure 2. Excision of the swelling was done with part of the septum and the upper lateral cartilage, preserving intact nasal mucosa. About 2-3 mm of cartilage as a safety margin was included with the mass. The specimen was sent for histopathological examination.

Reconstruction of both the septal and upper lateral cartilage defects was done by use of conchal cartilage graft, spreader and strut cartilage grafts were done. Nasal pack of Vaseline gauze was introduced for 24 h, and the collumellal stitches were removed on day 5 post operatively.

The histopathology of the specimen showed rhinoscleroma, fibrotic infiltrative stage.

**Figure 3:**
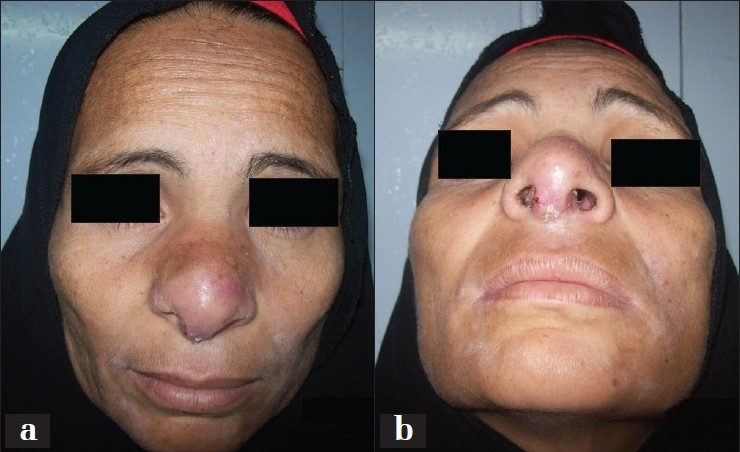
Post operative views of the patient at 6 months

The patient received antimicrobial drug in the form of ciprofloxacin, 750 mg twice daily, for 1 week post operatively. Follow up for 3 weeks, 3 months, and 6 month post operative revealed a good functional and aesthetic outcome with no recurrence [Figure 3].

## DISCUSSION

Rhinoscleroma is a specific chronic granulomatosis disease caused by enterobacteria of the Klebsiella family (*K. rhinoscleromatis*), a gram-negative diplobacillus localized electively in the upper airways, particularly the nasal fossae, leading to the term “rhinoscleroma”. Rhinoscleroma is a rare disease and consequently national and international epidemiological data are scarce. It has been reported in 168 countries, mostly from Central Europe, extending into Ukraine and around the Black and Caspian Seas.[5] Regions with high endemicity (e.g., Guatemala,[6] El Salvador,[7] Mexico,[8] Colombia,[9] and Egypt[10]) have hundreds of reported cases. Rhinoscleroma is more frequent among people in the second or third decades of life, living in crowded conditions, rural areas, with poor hygienic and nutritional conditions such as iron deficiency anemia. A definite female preponderance with a female:-male ratio of 13:1 is noted. The presence of *K. rhinoscleromatis* is not enough for the development of the disease, as contact of the patient with healthy individuals for many years may not necessarily bring about the infection in the latter. This has led to the suggestion that susceptibility of the host is important in the development of the disease. Impaired cellular immunity seems to be a major culprit.[11] Cellular immunity is impaired in patients with rhinoscleroma; however, their humoral immunity is preserved.

Rhinoscleroma usually affects the nasal cavity, but lesions associated with rhinoscleroma may also affect the larynx, nasopharynx, oral cavity, paranasal sinuses, or soft tissues of the lips, nose, trachea, and bronchi.[3] The disease initially involves the nasal mucosa but may progress to any part of the airway. The disease less commonly affects the cartilaginous framework of the nose,[5] as in our case of septal rhinscleroma with healthy mucosa, apart from mucosal and inferior turbinate hypertrophy.

Untreated rhinoscleroma tends to progress slowly over many years, characterised by periods of remissions and relapses. During the earlier stage of the disease, adherent white plaques and granulations of different sizes may be seen in the airway. A culture of this material will have a high yield for the organism.[6]

Although our patient was diagnosed at a later stage due to her low socioeconomic status and neglect, she did not give a history of crustation or discharge. The nasal septum is an uncommon site for rhinoscleroma, and the patient presented a history irrelevant to the disease, but the histopathological examination surprisingly diagnosed it as rhinoscleroma.

Open tip rhinoplasty was a good approach for excising the swelling and also for the reconstruction to be done with ease.
